# Covid‐19 in patients with hematological and solid cancers at a Comprehensive Cancer Center in Germany

**DOI:** 10.1002/cam4.3460

**Published:** 2020-09-15

**Authors:** Khalid Shoumariyeh, Francesca Biavasco, Gabriele Ihorst, Siegbert Rieg, Alexandra Nieters, Winfried V. Kern, Cornelius Miething, Justus Duyster, Monika Engelhardt, Hartmut Bertz

**Affiliations:** ^1^ Department of Medicine I Medical Center ‐ University of Freiburg, Faculty of Medicine, University of Freiburg Germany; ^2^ German Cancer Consortium (DKTK), Partner Site Freiburg Freiburg Germany; ^3^ Clinical Trials Unit Freiburg, Faculty of Medicine University of Freiburg Germany; ^4^ Division of Infectious Diseases, Department of Medicine II Medical Center ‐ University of Freiburg, Faculty of Medicine, University of Freiburg Freiburg Germany; ^5^ Institute for Immunodeficiency Medical Center University of Freiburg Freiburg Germany

**Keywords:** cancer management, epidemiology, epidemiology and prevention, viral infection

## Abstract

**Background:**

Patients with cancer are considered a high‐risk group for viral pneumonia, with an increased probability of fatal outcome. Here, we investigated the clinical characteristics and outcome of patients with solid and hematological cancers and concomitant Covid‐19 at a Comprehensive Cancer Center in a Covid‐19 hotspot area in Germany.

**Methods:**

We performed a retrospective single center cohort study of 39 patients with hematological and solid cancers who were hospitalized at the University Hospital Freiburg for Covid‐19. Using univariate and multivariate Cox regression models we compared time to severe events and overall survival to an age‐matched control cohort of 39 patients with confirmed Covid‐19 without a cancer diagnosis.

**Results:**

In the cancer cohort 29 patients had a diagnosis of a solid tumor, and 10 had a hematological malignancy. In total, eight patients (21%) in the cancer and 14 patients (36%) from the noncancer cohort died during the observation period. Presence of a malignancy was not significantly associated with survival or time to occurrence of severe events. Major influences on mortality were high IL‐6 levels at Covid‐19 diagnosis (HR = 6.95, *P* = .0121) and age ≥ 65 years (HR = 6.22, *P* = .0156).

**Conclusions:**

Compared to an age‐matched noncancer cohort, we did not observe an association between a cancer diagnosis and a more severe disease course or higher fatality rate in patients with Covid‐19. Patients with a hematological malignancy showed a trend towards a longer duration until clinical improvement and longer hospitalization time compared to patients with a solid cancer. Cancer per se does not seem to be a confounder for dismal outcome in Covid‐19.

## Graphical abstract

In this retrospective single center cohort study at a Comprehensive Cancer Center in Germany we did not observe an association between a cancer diagnosis and a more severe disease course or higher fatality rate in patients with Covid‐19 when compared to an age‐matched control cohort of patients with confirmed Covid‐19 without a cancer diagnosis.

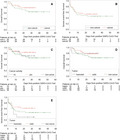

## 1 BACKGROUND

The novel severe acute respiratory syndrome coronavirus 2 (SARS‐CoV‐2) was discovered in the Chinese city of Wuhan in December 2019 where it quickly led to a severe outbreak.^1^, ^2^ In just a few weeks it evolved into a worldwide pandemic.^3^ Around 15%‐20% of infected people develop severe symptoms requiring hospitalization, and 3%‐6% of patients succumb to Covid‐19, most commonly due to acute respiratory distress syndrome (ARDS).^4^, ^5^ Currently, no specific treatment for Covid‐19 or vaccine against SARS‐CoV‐2 is available, while trials evaluating pharmacological interventions are ongoing.

Risk factors associated with adverse outcome among hospitalized patients have been proposed, among them are age > 65 years, male gender, obesity and presence of substantial comorbidities (cardiovascular disease, diabetes mellitus, respiratory disease, chronic kidney disease (CKD), liver disease, immunosuppression).^6^ Inflammation plays an intricate role in the pathophysiology of SARS‐CoV‐2 infection. In severe cases an overwhelming inflammatory host response to the virus leads to a release of vast amounts of cytokines, causing the clinical symptoms of sepsis with subsequent multi‐organ failure and death.^7^ Due to the decisive role of the host immune response and hyperinflammation several immunosuppressive therapies are being investigated to dampen immune‐mediated tissue damage in severe cases of Covid‐19.^8^

Patients with cancer are usually considered a high‐risk group for viral pneumonia, with an increased probability of severe infection and fatal outcome than in noncancer patients.^9^ The malignant disease itself and anticancer treatments, such as cytotoxic chemotherapy, novel anticancer agents, radiation therapy and surgery are known to lead to immunosuppression.^10^

So far, the immune response of patients with a malignancy to a concurrent SARS‐CoV‐2 infection has not been investigated. It is unclear, whether the immunosuppressant state in patients with malignancies predisposes them to a more severe Covid‐19 disease course. First reports from China indicated that cancer patients with Covid‐19 may have a higher risk of a severe disease course and less favorable outcome compared to noncancer patients with Covid‐19.^11^, ^12^, ^13^, ^14^ However, sample sizes were small and patient cohorts consisted mainly of patients with solid tumors.^11^, ^12^

Recently, two reports from China and one from the UK described larger multi‐center studies on cancer patients with Covid‐19.^15^, ^16^, ^17^ Tian et al analyzed risks for disease severity in 232 hospitalized patients with cancer admitted to nine hospitals in Wuhan.^15^ They reported that cancer patients with Covid‐19 had a higher risk of severe disease courses than a matched cohort without cancer.^15^ Yang et al analyzed factors associated with mortality in 205 patients with cancer and SARS‐CoV‐2 infection in nine hospitals within the Hubei region.^16^ In their multivariate regression analysis receiving chemotherapy within 4 weeks before symptom onset of SARS‐CoV‐2 infection and male sex were associated with death during hospitalization.^16^ Lee et al investigated the role of anticancer treatment on outcome of Covid‐19 in 800 cancer patients in a prospective multi‐centre observational study.^17^ Notably, mortality in cancer patients with Covid‐19 was not influenced by cytotoxic chemotherapy, but age, gender and comorbidities.^17^

Despite these existing studies, it remains unclear, whether cancer and immunosuppression in these patients as well as cytotoxic chemotherapy are relevant risks for and during Covid‐19 infections and why there are discrepancies in outcome between prior reports, which may result from different resources, health care systems and/or patient‐specific factors.

We therefore analyzed the outcome in cancer patients with concomitant Covid‐19 in a hotspot area in Germany, where case fatality rates due to Covid‐19 were relatively low and the SARS‐CoV‐2 pandemic did not overwhelm the health care system, as was the case in some areas of Europe and the US. As of 1 May 2020 a total of 160.758 cases with SARS‐CoV‐2 infection had been confirmed in Germany with 6.481 Covid‐19‐related deaths registered. In this single center study we assessed clinical endpoints in cancer patients (with both solid and hematological malignancies) with concomitant SARS‐CoV‐2 infection treated at our Comprehensive Cancer Center (CCC) of the University Hospital of Freiburg (UHF). Our goal was to determine the influence of cancer on morbidity and mortality of hospitalized Covid‐19 cancer patients compared to age‐matched hospitalized noncancer Covid‐19 patients.

## 2 METHODS

### 2.1 Study design and patient selection

We performed a retrospective single center cohort study: from 27 February 2020 to 10 April 2020 hospitalized patients at the UHF with an active hematological, solid cancer or cancer in remission, and concomitant SARS‐CoV‐2 infection confirmed by reverse‐transcriptase polymerase chain reaction (RT‐PCR) assay were included. In total 39 patients meeting the criteria were enrolled. For the control cohort, 39 age‐matched hospitalized patients with confirmed Covid‐19 from the same time span without a cancer diagnosis were recruited. Patients gave written informed consent and the study was conducted in accordance with the tenets of the Declaration of Helsinki.

### 2.2 Study endpoints and assessment

For our analysis we divided the two SARS‐CoV‐2 positive groups into four cohorts: (a) patients without cancer (“noncancer cohort”, n = 39), (b) patients with a current diagnosis of cancer or cancer in remission (“cancer cohort”, n = 39), the latter group was further subdivided into (c) patients with a solid tumor (“solid cohort”, n = 29), and (d) patients with a hematological malignancy (“hematological cohort”, n = 10).

Analyzed endpoints included requirement for oxygen support, time to transfer to the intensive care unit (ICU), time to clinical improvement and overall survival (OS), duration of hospitalization and time to severe events (TTS).^13^

For evaluation of “requirement for oxygen support” we defined four groups: (a) ambient air, (b) nasal low‐flow oxygen therapy, (c) nasal high‐flow (HF) oxygen therapy/noninvasive ventilation (NIV), and (d) invasive mechanical ventilation including extracorporeal membrane oxygenation (ECMO). “Ordinal Scale for Clinical Improvement” designed by a World Health Organization (WHO) committee to measure illness severity over time.^19^, ^20^

Clinical improvement was defined by a decrease of at least 2 points from baseline on the modified WHO ordinal scale or hospital discharge, or both. Only patients who did not die during the observation period were included for the analyses of time to clinical improvement and duration of hospitalization.

OS was defined as time from the date of a positive RT‐PCR for SARS‐CoV‐2 to death from any cause. TTS was defined as time from positive RT‐PCR date to transfer to ICU, mechanical ventilation, or death, whichever occurred first. For OS and TTS, patients without observation of the event of interest were considered as a censored observation at the data cutoff date 20 April 2020. For 11 patients with a positive RT‐PCR obtained 0‐2 days after transfer to ICU or start of mechanical ventilation, 0.5 days was inserted for OS/TTS. In one patient, mechanical ventilation was started prior to transfer to ICU, thus TTS and time to ICU transfer nearly coincided.

### 2.3 Data acquisition and statistical analysis

Laboratory values at initial diagnosis of Covid‐19, during the disease course and clinical outcomes were collected from medical records. Data collected through 20 April 2020 were used for analysis. Data were analyzed using SAS statistical software version 9.2 (SAS Institute Inc). Group comparisons were performed with Wilcoxon two sample tests for continuous variables, Fisher's exact test or chi‐square tests for binary variables, Mantel‐Haenszel chi‐square tests for ordered categorical variables, and logrank tests for time‐to‐event variables. A *P*‐value < .05 was defined as statistically significant. OS and TTS rates were estimated and displayed using the Kaplan‐Meier method. Univariate and multivariate Cox regression models were used in order to compare OS and TTS in cancer and noncancer patients. Age ≥ 65 years, renal disease, pulmonary disease, presence of two or more comorbidities, smoking, male sex, CRP ≥ 100 mg/dL, interleukin 6 (IL‐6) ≤ 58.3 pg/mL (median), and lymphocytes ≤ 0.695 × 10^9^/L (median) were considered as potential relevant prognostic factors. Factors that were univariately significant were considered in a multivariate model and were subjected to variable selection (backward elimination with *P* = .15). Results are presented as hazard ratios (HRs) with two‐sided 95% confidence intervals (95% CI). Missing values for IL‐6 and lymphocytes were defined as a separate class as a possibility to include these patients in the regression analyses.

## 3 RESULTS

### 3.1 Demographic and clinical characteristics

In the greater area of Freiburg in the southwest state of Baden‐Wuerttemberg in Germany with 492.000 inhabitants a total of 1569 SARS‐CoV‐2 infections were registered as of 10 April 2020. Of these, 167 patients were hospitalized at the UHF due to Covid‐19. In total, 52 (31%) of the 167 patients required intensive care treatment, while 23 (14%) patients succumbed to the disease.

Among the 167 patients who were hospitalized at the UHF from 27 February 2020 until 10 April 2020, a total of 39 patients (23.4%) had an active hematological (n = 10, 6%) or solid cancer (n = 14, 8.4%) or a cancer in remission (n = 15, 9%). Clinical characteristics of these patients and of age‐matched controls (n = 39) of hospitalized patients with a positive RT‐PCR test for SARS‐CoV‐2, but no history or current diagnosis of cancer, are listed in Table 1.

**TABLE 1:** Clinical characteristics of the different cohorts

Patients characteristics	Cancer cohort (n = 39)	NonCancer cohort (n = 39)	*P*‐value	Solid tumors (n = 29)	Hematological tumors (n = 10)	*P*‐value
Age @ Covid‐19 diagnosis; median (range)	73 (54‐95)	76 (45‐96)	.3709	73 (54‐95)	71 (55‐85)	.8353
< 65 yrs; n	12	12		8	4	
65‐75 yrs; n	9	6		8	1	
> 75 yrs; n	18	21		13	5	
Age at diagnosis of cancer; median (range)	66 (45‐83)	—		67 (48‐82)	60 (45‐83)	.3276
Active:nonactive disease; n	24:15			15:14	9:1	
f: m; n	17:22	14:25	.6439	13:16	4:6	1.0000
Nosocomial: community acquired	14:20	16:17	.6267	11:14	3:6	.7401
Comorbidities						
Cardiovascular; n	30	34	.3768	23	7	.6686
Diabetes; n	5	9	.3768	4	1	1.0000
Asthma/COPD; n	3	11	.0362	2	1	1.0000
CKD; n	5	15	.0183	4	1	1.0000
Rheumathologic disease; n	3	3	1.0000	3	0	.5558
Chronic liver disease; n	1	3	.6151	0	1	.2564
≥ 2 organ involvements; n	11	23	.0115	8	3	1.0000
Sum of organ involvements; median	1	2	.0055	1	1	.7314
History of smoking; n	6	9	.4998	6	0	.1141
Cancer treatment in last 4 weeks						
Chemo/radio/immunotherapy; n	9	—		6	3	
Surgery only; n	5	—		5		
None; n	25	—		18	7	
Cancer treatment in last 4 y to 4 wks						
Chemo/radio/immunotherapy; n	3	—		2	1	
Surgery only; n	2	—		2		
None; n	20	—		14	6	
Type of cancer						
Uro‐gynecological; n	16	—				
Lung; n	5	—				
Head&neck/Skin; n	5	—				
GI; n	3	—				
Stage IV; n	10	—				
Lymphoma/Myeloma; n	7	—				
Leukemia/PNH; n	3	—				

Abbreviations: CKD, chronic kidney disease; COPD, chronic obstructive pulmonary disease; GI, gastrointestinal; n, number of patients; PNH, paroxysmal nocturnal hemoglobinuria.

In our entire patient population (Covid‐19 cancer and noncancer cohort; n = 78) the median age was 76 years, 60% were male and 19% had a smoking history. Cardiovascular comorbidities were the most prevalent, followed by CKD and diabetes mellitus (DM).

In the solid cancer cohort, 10 patients had a metastatic tumor, and uro‐gynecological malignancies (including prostate, breast and urothelial cancer) accounted for 41% of all malignancies. In the hematological cohort, 18% had lymphoma/myeloma, making it the most common hematological neoplasms. More than 60% (24/39) of the cancer patients had an active cancer disease at the time of Covid‐19 diagnosis. Fourteen patients (36%) received anticancer treatment (cytotoxic chemotherapy, immunotherapy, radiation or surgery) for their malignancy in the last 4 weeks prior to diagnosis of Covid‐19. Patients with cancer vs without did not show a higher prevalence of nosocomial‐acquired Covid‐19 with 14/39 (36%) and 16/39 (41%), respectively (*P* = .63).

### 3.2 Subgroup analysis and comparisons of cancer vs noncancer controls with Covid‐19 infection

No significant difference regarding median age at the time of Covid‐19 diagnosis between cancer and noncancer controls was observed (73 vs 76 years, *P* = .37), gender distribution (*P* = .64), smoking history (*P* = .50), presence of cardiovascular comorbidities (*P* = .38), presence of DM (*P* = .38), presence of rheumatologic diseases (*P* = 1.00) or presence of chronic liver disease (*P* = .61), thus both groups were well‐matched and well comparable.

Pulmonary diseases (chronic obstructive pulmonary disease (COPD), asthma) and renal impairment/CKD were slightly more prevalent in the noncancer vs cancer cohort (11/39 vs 3/39, *P* = .0362; and 15/39 vs 5/39, *P* = .0183, respectively).

Considering cancer patients alone, no significant differences between solid and hematological malignancies in age, gender, smoking history or comorbidities were found (Table 1).

### 3.3 Laboratory parameters

Blood counts at diagnosis of Covid‐19 showed normal leukocyte, neutrophil and thrombocyte counts among the different cohorts (Table 2).

**TABLE 2:** Laboratory findings in the different cohorts

Laboratory parameter	Cancer cohort (n = 39)	Noncancer cohort (n = 39)	*P*‐value	Solid tumors (n = 29)	Hemato‐logical tumors (n = 10)	*P*‐value
Leukocytes (10^9^/L)	5.85	6.33	.4263	6.28	5.63	.5987
Platelets (10^9^/L)	175	172	.8990	179	142	.2476
Hemoglobin (g/dL)	10.9	12.4	.2811	11.6	9.9	.1428
Neutrophils (10^9^/L)	4.67	4.81	.7454	5.04	3.71	.6168
Lymphocytes (10^9^/L)	0.57	0.85	.0519	0.78	0.39	.0588
D‐Dimers (mg/L)	2.83	1.02	.1112	3.94	1.46	.1076
max. D‐Dimers (mg/L)	4.03	6.03	.8840	3.68	4.41	.6331
LDH (IU/L)	328	344	.5132	318	339	.4120
max. LDH (IU/L)	396	465	.2744	336	532	.1006
CRP (mg/L)	68	64	.9352	51	96	.2005
max. CRP (mg/L)	127	168	.1017	87	181	.1078
PCT (ng/mL)	0.13	0.2	.0062	0.12	0.14	.3197
max. PCT (ng/mL)	0.22	0.77	.0110	0.19	0.43	.1076
IL‐6 (pg/mL)	47.8	82.2	.0634	47.8	50.2	.5063
max. IL‐6 (pg/mL)	64.5	118	.2481	59.5	274.5	.0298
GFR (mL/min)	70	48	.0260	70	68	.5611

Abbreviations: CRP, C‐reactive protein; GFR, glomerular filtration rate; IL‐6, interleukin 6; LDH, lactate dehydrogenase; PCT, procalcitonin.

In cancer vs noncancer patients, mild anemia (median hemoglobin (Hb): 10.9 vs 12.4 g/dl,) and lymphopenia (median 0.57 vs 0.85 × 10^9^/L; *P* = .0519) were present, respectively (Table 2).

D‐dimers, lactic dehydrogenase (LDH) and C‐reactive protein (CRP), as parameters of systemic inflammation, were elevated at admission and during hospitalization, but did not reveal significant differences between cancer and noncancer patients (Table 2).

Procalcitonin (PCT) was used as an indicator for bacterial superinfection. Median PCT at admission was low (<0.5 ng/mL) in all cohorts, though with statistically significant higher levels in the noncancer vs cancer cohort (0.2 and 0.13 ng/mL, respectively; *P* = .0062). Furthermore, a greater increase of PCT during hospitalization was observed in the noncancer vs cancer cohort (median of maximum values: 0.77 ng/mL vs 0.22 ng/mL; *P* = .0110; Table 2).

In our patients IL‐6 at admission was variable but showed no significant differences between the cancer vs non‐cancer cohort (median: 47.8 pg/mL vs 82.2 pg/mL, *P* = .0634).

Consistent with the higher prevalence of CKD in the noncancer cohort, the glomerular filtration rate (GFR) was significantly lower than in cancer patients (48 vs 70 mL/min; *P* = .00260; Table 2).

Focusing on hematological and solid cancer patients alone, a slightly decreased platelet count (142 × 10^9^/L) and more pronounced lymphopenia (0.39 × 10^9^/L) at the time of Covid‐19 diagnosis were present in patients with hematological malignancies. Moreover, these patients showed a statistically significant greater increase in IL‐6 during hospitalization than the solid cancer cohort (median maximum value: 274.5 pg/mL vs 59.5 pg/mL; *P* = .0298; Table 2).

### 3.4 Treatment

At our CCC UHF‐institution, patients who developed severe Covid‐19‐associated pneumonia with respiratory insufficiency or had potential risk factors (cardiovascular or pulmonary comorbidities, immunosuppression, age > 60 years), received hydroxychloroquine and lopinavir/ritonavir for 5 and 7 days, respectively. In our study population, 20 cancer and 21 noncancer patients received specific Covid‐19 treatment. Of these, 12 cancer and 15 noncancer patients received the above antiviral treatment, with a similar distribution between both cohorts (31% and 38% respectively; *P* = .72). A nonsignificant difference between solid and hematological tumor patients with regard to antiviral treatment was notable (8/29 (28%) and 4/10 (40%) respectively, *P* = .0766). Four cancer and two noncancer patients received hydroxychloroquine only and 3 cancer patients received steroids with or without antiviral treatment. Only one cancer patient received hydroxychloroquine/oseltamivir and two non‐cancer patients lopinavir/ritonavir alone. In ARDS patients, tocilizumab was used in two noncancer patients (Table 3). Additional antibiotic therapy was given to the majority of patients (cancer: 25/39 (64%); noncancer: 32/39 (82%)). Two patients from the hematological cohort received granulocyte colony stimulating factor due to transient leukopenia and persistent infection (Table 3).

**TABLE 3:** Treatment modalities and causes of death in the different cohorts

Treatment and ICU support in n of pts	Cancer cohort (n = 39)	Noncancer cohort (n = 39)	Solid tumors (n = 29)	Hematological tumors (n = 10)
Antibiotics	25	32	18	7
Filgrastim	2	0	0	2
Supportive Treatment/Palliation	4	9	2	2
COVID‐19 therapy				
Hydroxicloroquine + Lopinavir/Ritonavir	12	15	8	4
Hydroxicloroquine alone	4	2	3	1
Other^1^	4	4	1	1
None	19	18	17	2
Oxygen therapy				
Ambient air	12	12	11	1
Low flow oxygen	12	16	10	2
High flow oxygen/NIV	4	0	0	4
Mechanical ventilation/ECMO	11	11	8	3
Outcome				
ICU	16	14	9	7
Discharged, n	18	15	15	3
Deceased, n	8	14	6	2
Duration of hospitalization, median days	13.5	9	13	21
Time to clinical improvement, median days	13	9	11.5	18.5
Causes of death				
Hypoxia	2	7	1	1
ARDS	3	2	2	1
Sepsis	2	3	2	0
Other^2^	1	2	1	0

Abbreviations: ARDS, acute respiratory distress syndrome; ECMO, extracorporeal membrane oxygenation; ICU, intensive care unit; n, number of patients; NIV, noninvasive ventilation; pts, patients.

^1^ Hydroxychloroquine + Oseltamivir, Steroids, Tocilizumab.

^2^ Arrythmia, intracerebal bleeding.

### 3.5 Need for intensive care support

Almost 70% of patients received one form of oxygen therapy during hospitalization. No difference in intensity of oxygen support between cancer and noncancer cohorts was apparent (ambient air: 31% vs 31%, nasal low‐flow: 31% vs 41%, nasal high‐flow or NIV: 10% vs 0%, invasive mechanical ventilation 28% vs 28%, respectively; Table 3). Four patients (two from each cancer/noncancer cohort) received ECMO treatment. There was no association of cancer vs noncancer patients for ICU requirements/intensive care treatment (41% vs 36%, respectively; Table 3).

When comparing solid and hematological tumors, we observed that the latter group required nasal high‐flow oxygen therapy and invasive mechanical ventilation more often (ambient air: 10% vs 38%, nasal low‐flow: 20% vs 34%, nasal high‐flow or NIV: 40% vs 0%, invasive mechanical ventilation: 30% vs 28%; Table 3). In line, transfer to the ICU was increased for the hematological vs solid tumor cohort (70% vs 31%, respectively; Table 3).

### 3.6 Survival

**FIGURE 1:**
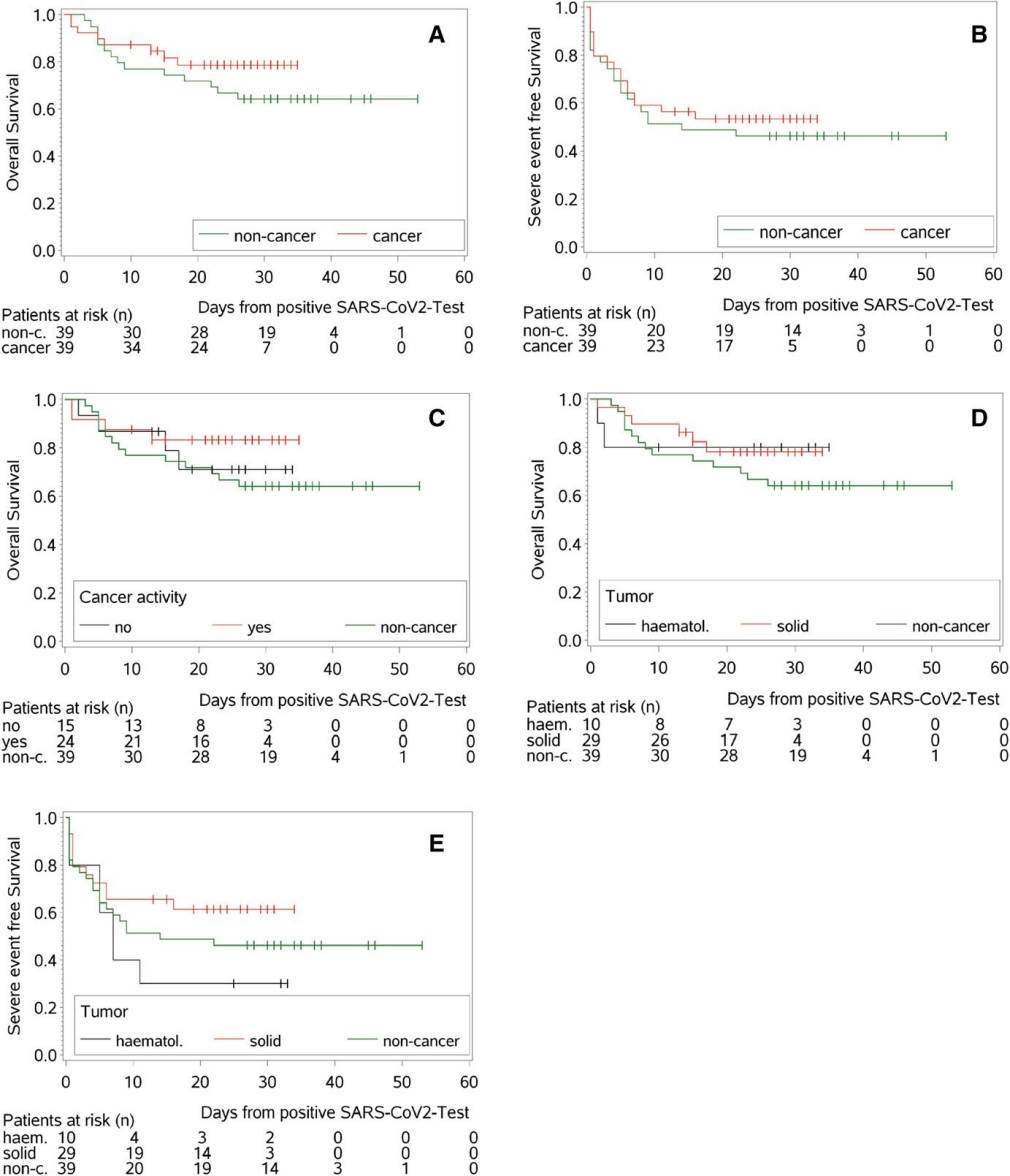
Kaplan‐Meier plots for overall and severe event free survival. A, Kaplan‐Meier estimates for overall survival probability for the cancer and noncancer cohort. B, Kaplan‐Meier estimates for overall survival for the hematological, solid and the noncancer cohort. C, Kaplan‐Meier estimates for overall survival probability based on cancer activity. D, Kaplan‐Meier estimates for severe event free survival for the cancer and noncancer cohort. E, Kaplan‐Meier estimates for severe event free survival for the hematological, solid and noncancer cohort

At time of admission, 10% of the cancer and 23% of the noncancer patients dismissed the option of intensive care treatment, and were therefore treated with best supportive care in case of clinical deterioration. Eight cancer and 14 noncancer patients died during hospitalization (21% vs 36%, respectively; Table 3). The causes of death are listed in Table 3. Kaplan‐Meier estimates showed survival at 30 days [30dOS] for the cancer vs noncancer cohort of 78.6% (95% CI: 61.6%‐88.7%) vs 64.1% (95% CI: 47.0%‐76.9%, respectively; Figure 1A).

No significant difference was observed in terms of OS between solid tumor and hematological patients (30dOS: 78.2% vs 80.0%, *P* = .95; Figure 1B). Since the cancer cohort included patients with active cancer and some with cancer in remission, we investigated whether patients with active cancer showed a worse outcome: notably, the latter did not have a more dismal OS compared to those without cancer (30dOS active cancer: 83.1% (95% CI: 61.0%‐93.3%) vs nonactive cancer: 70.9% (95% CI: 39.5%‐88.1%) vs noncancer: 64.1% (95% CI: 47.0%‐76.9%), Figure 1C). To evaluate the severity of Covid‐19 in our population we analyzed the duration of hospitalization, TTS and the time to achievement of clinical improvement. Again, cancer and noncancer cohorts showed no differences in severity of Covid‐19 (Figure 1D). Patients with hematological malignancies showed a nonsignificant trend for earlier occurrence of severe events compared to solid tumor patients (severe event free survival for hematological cohort = 30% vs severe event free survival for solid cohort = 61.4%, Figure 1E), had a longer duration of hospitalization (median hematological = 21 days vs median solid cohort = 13 days), and required a longer time to achieve a clinical improvement (median hematological cohort = 18.5 days vs median solid cohort = 11.5 days).

### 3.7 Prognostic factors: uni‐ and multivariate analysis

In a univariate Cox proportional hazards regression analysis for all patients, age ≥ 65 years, CRP ≥ 100 mg/dL, elevated IL‐6, renal impairment/CKD and presence of ≥ 2 organ comorbidities were significantly associated with a higher probability of death (Figure S1A, Table S1). Using these factors, we performed a multivariate Cox regression analysis for OS with backward elimination. Adjusted for the remaining factors, the HR of cancer vs noncancer patients was close to 1 (HR = 0.99, 95%CI 0.40‐2.43, *P* = .98), indicating a nearly identical risk of dying of Covid‐19 for both groups. Elevated IL‐6 levels represented the most prominent prognostic factor (HR = 7.48, 95% CI 2.13‐26.3, *P* = .0017; Table 4).

**TABLE 4:** Multivariate Cox regression analysis for OS and severe event free survival

	HR	95% CI	*P*‐value
Overall survival			
Cancer	0.986	0.400‐2.431	.9757
≥ 2 organ comorbidities	1.521	0.416‐5.560	.5262
Age ≥ 65 yrs	6.343	1.448‐27.787	.0142
IL‐6 > median value	7.476	2.125‐26.301	.0017
CKD	2.0.029	0.836‐4.922	.1176
Severe event free survival			
Cancer	0.870	0.459‐1.648	.6692
≥ 2 organ comorbidities	1.261	0.607‐2.618	.5343
IL‐6 > median value	5.440	2.200‐13.448	.0002
Lymphocyte count > median value	0.629	0.273‐1.447	.2750

Abbreviations: CKD, chronic kidney disease; IL‐6, interleukine 6; yrs, years.

For TTS, CRP ≥ 100mg/dL, elevated IL‐6, ≥ 2 organ comorbidities and decreased lymphocyte counts, were selected via univariate analyses (Figure S1B, Table S2) as possible relevant prognostic factors: after variable selection, an adjusted HR = 0.87 for cancer patients was observed (95% CI: 0.46‐1.65, *P* = .67; Table 4). Again, the most relevant prognostic factor was an elevated IL‐6 value (Table 4).

## 4 CONCLUSION

Compared to other countries of similar size and population, Germany has had fewer absolute numbers of hospitalized patients and deaths attributed to Covid‐19. Thus, an early and adequate response to the pandemic permitted health care providers to adjust their strategies preventing overwhelming of ICU capacities.

To date, no clear evidence‐based guidelines in regard to the management of cancer patients during the SARS‐CoV‐2 pandemic have been established and prior analysis have to be interpreted with caution due to geographical differences in health care capacities and treatment modalities, all factors influencing patient outcome during this pandemic. Yet, numerous guidelines, often specific to cancer entities, have been swiftly published to aid clinicians in managing Covid‐19 infected cancer patients.^21^, ^22^, ^23^ We therefore performed this retrospective cohort study in 39 cancer patients and an age‐matched cohort of noncancer patients, who were hospitalized with confirmed SARS‐CoV‐2 infection at our CCC in one of the Covid‐19 hotspots of Germany. Novelties of our analysis were the inclusion of hematological and solid tumor patients with SARS‐CoV‐2 infection and the careful comparison to age‐matched noncancer Covid‐19 patients from the same time span, to address whether cancer per se induced a substantial risk on the outcome of Covid‐19. Our data, though they have to be interpreted with caution due to the small sample size, indicate that cancer patients receiving required therapy for severe Covid‐19, including intensive care treatment, mechanical ventilation and ECMO, have the same probability of survival as noncancer patients (HR = 0.99, 95%CI 0.40‐2.43, *P* = .98), thus that cancer per se does not bear a substantial risk for a dismal outcome.

As of April 20, eight cancer patients hospitalized at the CCC UHF for Covid‐19 had died, accounting for a lethality among hospitalized Covid‐19 cancer patients of 20.5%. In comparison, 36% of the noncancer cohort patients succumbed to Covid‐19. In our age‐matched noncancer cohort, comorbidities affecting both lung and kidney were more prevalent than in the cancer cohort. After adjusting for this confounder, we could not detect a difference in OS between cancer and noncancer cohorts. The numbers for lethality are in line with a report from Zhou et al reporting on the clinical course and mortality of inpatients with Covid‐19 in Wuhan. In their analysis 28.3% of 191 hospitalized patients died due to Covid‐19.^23^ In a UK‐wide observational cohort study of 20 133 hospitalized Covid‐19 patients a mortality rate of at least 26% was reported.^24^ More recently, in a retrospective international multi‐center study conducted by a cancer consortium from the US, Canada and Spain evaluating the outcome of 928 cancer patients during the Covid‐19 pandemic a crude mortality rate for hospitalized patients of 23% was revealed.^25^ Of note, patients included by the Canadian colleagues had the highest admission rate to the hospital, while at the same time displaying the lowest rate of deaths of any geographical subgroup.^25^

Notably, in our study, both cancer and noncancer patients required transfer to the ICU at similar rates (35.9% and 41%, respectively), whereas patients with a hematological malignancy more often required intensive care treatment (70% vs 30%) and more invasive ventilation (30% vs 27.5%) vs patients with solid tumors. Other published studies investigating Covid‐19 in cancer patients have similarly reported a higher rate of ICU admission and need for invasive ventilation in patients with hematological malignancies.^12^, ^27^

Furthermore, hematological patients showed a trend towards earlier occurrence of severe events, a longer duration of hospitalization, and longer time to achieve a clinical improvement compared to patients with a solid cancer. Additionally, maximum levels of the proinflammatory cytokine IL‐6 were higher in patients with hematological malignancies compared to solid tumors, indicating a more pronounced inflammatory response in these patients, although IL‐6 levels at diagnosis of Covid‐19 were comparable between those two cohorts and the noncancer patients. An overwhelming inflammatory host response to SARS‐CoV‐2, in which vast amounts of cytokines are released, has been proposed as a hallmark of severe cases of Covid‐19.^27^ This cytokine release syndrome eventually results in clinical symptoms resembling sepsis with multi‐organ failure and ARDS.^27^ Several studies have shown an association between high IL‐6 levels and worse outcome in Covid‐19 patients.^28^ Our sample size was too small to elucidate which hematological patients are at risk for developing a hyperinflammatory state. Further research efforts are warranted to evaluate whether IL‐6 blockade via tocilizumab can alter the disease course in patients with hematological malignancies and Covid‐19.

In a multivariate Cox regression analysis, age ≥ 65 years (HR = 6.34, 95%CI 0.42‐5.56, *P* = .0142) and elevated IL‐6 levels (HR = 7.45, 95% CI 2.125‐26.30, *P* = .002) at diagnosis were significantly associated with worse OS and severe event free survival (HR = 5.44, 95% CI 2.200‐13.448, *P* = .002). This is in line with recently published studies investigating the clinical characteristics in a general population with Covid‐19.^23^ Cancer or other comorbidities did not show a significant association with severe disease course or worse outcome (Table 4).

Our study was limited by the fact that this was a retrospective analysis of a single institution, and therefore, data have to be interpreted with caution. At the time of the first Covid‐19 cases at our hospital a multi‐center approach did not seem feasible due to the emergency of the SARS‐CoV‐2 outbreak. Relevant were, however, the well‐matched comparison of cancer and noncancer patients, treated in a European, heavily Covid‐19‐striken hotspot area that allocated plenty of resources to Covid‐19 care and avoided accumulation of large patient crowds.

To account for the heterogeneity of different cancer entities and their different biological behavior, as well as diverse treatment strategies, future studies on the influence of cancer on Covid‐19 disease severity and mortality need to be prospective, multi‐center and international. Additionally, more detailed analysis of risk factors attributed to the host immune response and associated with worse outcome in cancer patients undergoing active treatment are required. To date, it has not been elucidated whether the impaired immune response in some cancer patients might prevent hyperinflammation and thus severe disease courses in Covid‐19.

In summary, we performed detailed statistical analyses of cancer patients with concomitant Covid‐19 at a CCC in Germany. We compared this cohort with an age‐matched group of patients with Covid‐19, but without cancer. To the best of our knowledge, this is the first study assessing the outcome of hospitalized cancer patients with Covid‐19 in Germany. Contrary to previous reports from Chinese colleagues, we did not observe a greater disease severity or mortality of Covid‐19 in our cancer cohort.^12^, ^14^, ^15^, ^16^ Our data indicate that if cancer patients receive necessary supportive treatment for Covid‐19, cancer itself does not seem to be an independent risk factor for dismal outcome, though, due to the small number of patients, our findings have to be interpreted with caution and need to be confirmed by larger studies. Patients with a hematological malignancy seem at risk for more severe disease courses, though this needs to be confirmed by larger multi‐center studies. Based on our data, postponing of chemotherapy or necessary surgery for cancer patients during the SARS‐CoV‐2 pandemic seems less required than initially presumed, especially in areas not overwhelmed by Covid‐19 patients.

## CONFLICT OF INTERESTS

The authors declare that they have no conflicts of interest.

## AUTHORS’ CONTRIBUTIONS

KS designed the study, analyzed and interpreted data and wrote the manuscript; FB designed the study, analyzed and interpreted data and wrote the manuscript; GI performed the statistical analyses; SR helped with the conception of the study and provided patient data; AN helped with the conception and design of the study and wrote the manuscript; WVK helped with the conception and design of the study; CM helped with the conception and design of the study and wrote the manuscript; JD designed the study and wrote the manuscript; ME designed the study and wrote the manuscript; HB designed the study and wrote the manuscript. All authors approved the final version of the manuscript.

## Supporting information

Fig S1

Table S1‐S2

## Data Availability

The data that support the findings of this study are available on request from the corresponding author. The data are not publicly available due to privacy or ethical restrictions.
