# Supplementary material for: Covid‐19 in patients with hematological and solid cancers at a Comprehensive Cancer Center in Germany
Source: Cancer Med. 2020 Sep 15;9(22):8412–22. doi: 10.1002/cam4.3460 (PMC7666742; doi:10.1002/cam4.3460)
Supplement: Supplementary file 1 — Fig S1 [file CAM4-9-8412-s001.pdf]

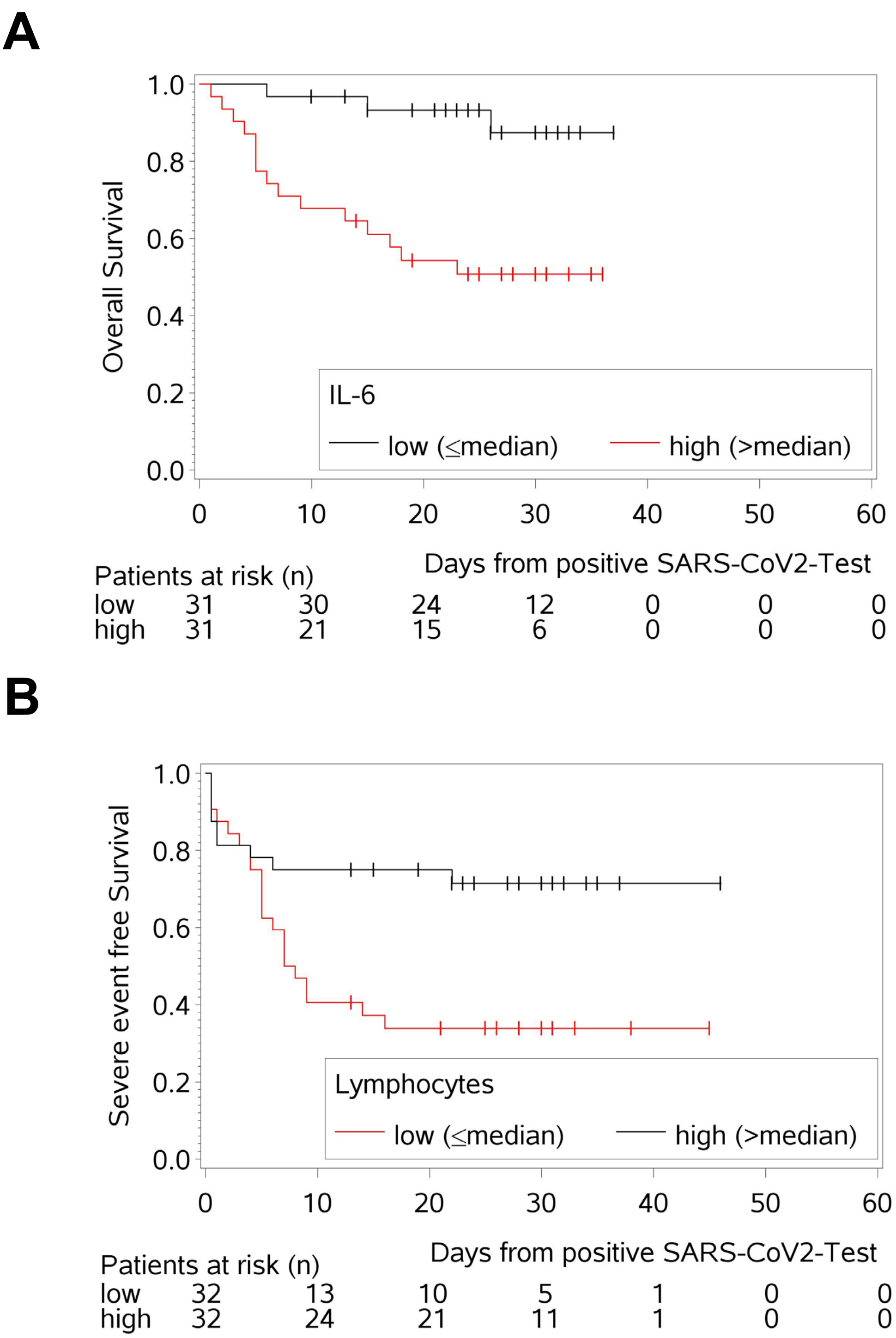


**Supplementary Figure 1.** A) Kaplan Meier estimates for overall survival based on IL-6 levels at diagnosis of Covid-19. B) Kaplan Meier estimates for severe event  free survival based on absolute lymphocyte counts at diagnosis of Covid-19.
