# Supplementary material for: Covid‐19 in patients with hematological and solid cancers at a Comprehensive Cancer Center in Germany
Source: Cancer Med. 2020 Sep 15;9(22):8412–22. doi: 10.1002/cam4.3460 (PMC7666742; doi:10.1002/cam4.3460)
Supplement: Supplementary file 2 — Table S1‐S2 [file CAM4-9-8412-s002.pdf]

Supplementary Table 1. Univariate Cox proportional hazards regression analysis for Overall Survival.

**Overall survival**	**HR**	**95% CI**	***p-value***
Cancer	0.609	0.255-1.455	0.2643
CKD	2.726	1.177-6.316	0.0193
Pulmonary disease	1.282	0.473-3.480	0.6252
≥2 organ comorbidities	3.121	1.271-7.663	0.0130
Smoking	1.153	0.351-3.785	0.8146
Age at Covid-19 ≥65	5.456	1.274-23.367	0.0222
Male	1.241	0.520-2.962	0.6258
CRP ≥100 mg/L	2.536	1.098-5.859	0.0294
IL-6 >median 58.3 pg/mL	6.257	1.809-21.638	0.0038
Lymphocytes ≤median 0.695x10^9/L	0.606	0.230-1.594	0.3098
Tumor Stage IV	0.916	0.178-4.723	0.9165
Solid Tumor	1.056	0.213-5.238	0.9469
Chemotherapy received ≤28 days before SARS-CoV-2-Test	0.560	0.113-2.779	0.4784

CKD, chronic kidney disease; HR, hazard ratio; CI, confidence interval.

Supplementary Table 2. Univariate Cox proportional hazards regression analysis for Severe event free Survival.

**Severe event free Survival**	**HR**	**95% CI**	***p-value***
Cancer	0.829	0.442-1.556	0.5592
CKD	1.456	0.747-2.836	0.2697
Pulmonary disease	1.218	0.560-2.651	0.6185
≥2 organ comorbidities	1.945	1.031-3.668	0.0399
Smoking	1.122	0.442-2.844	0.8088
Age at Covid-19 ≥65	1.154	0.575-2.319	0.6867
Male	1.492	0.766-2.907	0.2395
CRP ≥100 mg/L	3.028	1.603-5.721	0.0006
IL-6 >median 58.3 pg/mL	6.149	2.636-14.347	<.0001
Lymphocytes ≤median 0.695x10^9/L	0.370	0.169-0.810	0.0129
Tumor Stage IV	0.344	0.077-1.539	0.1626
Solid Tumor	2.054	0.793-5.316	0.1381
Chemotherapy received ≤28 days before SARS-CoV-2-Test	0.421	0.138-1.282	0.1277

CKD, chronic kidney disease; HR, hazard ratio; CI, confidence interval.
